# Systemic bevacizumab for high-output cardiac failure in hereditary hemorrhagic telangiectasia: an international survey of HHT centers

**DOI:** 10.1186/s13023-019-1239-6

**Published:** 2019-11-14

**Authors:** Hanny Al-Samkari, Hasan A. Albitar, Scott E. Olitsky, Marianne S. Clancy, Vivek N. Iyer

**Affiliations:** 1 Division of Hematology, Massachusetts General Hospital, Harvard Medical School, Zero Emerson Place Suite 118 Office 112, Boston, MA 02114 USA; 2 Department of Internal Medicine, Mayo Clinic, Rochester, MN USA; 3 Cure HHT, Monkton, MD USA; 4 Division of Pulmonary and Critical Care Medicine, Mayo Clinic, Rochester, MN USA

**Keywords:** Hereditary hemorrhagic telangiectasia, Osler-weber-Rendu, HHT, Bevacizumab, High-output cardiac failure, Heart failure, Arteriovenous malformation

## Abstract

**Background:**

Systemic bevacizumab is a novel targeted anti-angiogenic therapy for high-output cardiac failure (HOCF) in hereditary hemorrhagic telangiectasia (HHT) but published data is limited. This survey-based study measured physician-reported safety, effectiveness and current treatment practices for systemic bevacizumab in HHT-HOCF.

**Methods:**

A 27-item survey was sent to center directors of 31 international HHT Centers of Excellence.

**Results:**

Response rate was 74% with centers reporting 150 total patients receiving systemic bevacizumab for HHT-HOCF. Approximately two-thirds of centers had treated ≥5 patients. All centers utilize a 5 mg/kg dose for induction treatment and most administer 6 doses (range, 4–6) every 2 weeks, although maintenance regimens varied considerably. Center directors reported bevacizumab to be effective, with 55% reporting significant improvement in cardiac index and HOCF symptoms in most patients treated with bevacizumab, although normalization of cardiac parameters was uncommon. Adverse events were uncommon with three-quarters of centers reporting adverse event rates < 10%. Discontinuation for adverse events or ineffectiveness was rare. Bevacizumab was typically administered by hematologists and pulmonologists (50 and 39% of centers, respectively), with highly variable thresholds for initiation. Although half the centers reported difficulty with the insurance approval process, 70% of centers were ultimately able to obtain coverage for most or all of their patients.

**Conclusions:**

Systemic bevacizumab is a widely-used therapy for HHT-HOCF with reasonable safety and effectiveness. HHT centers appear to vary considerably in maintenance treatment practices and disease severity thresholds for initiation of bevacizumab in HHT-related HOCF.

## Introduction

Hereditary hemorrhagic telangiectasia (HHT, Osler-Weber-Rendu disease) is an autosomal dominant multisystem vascular disorder of disorganized angiogenesis [1]. HHT results in characteristic vascular abnormalities including arteriovenous malformations (AVMs) that affect multiple visceral organs (including liver, lungs and central nervous system) and bleeding telangiectasias involving the skin and mucus membranes [2, 3]. Clinical manifestations of HHT include recurrent epistaxis, gastrointestinal (GI) hemorrhage, iron deficiency anemia, high-output cardiac failure (HOCF), pulmonary hypertension, brain abscess, and others [4, 5].

Hepatic AVMs affect up to 75% of HHT patients, the majority of which are asymptomatic [6–8]. HHT-associated hepatic AVMs can result in 3 different types of intrahepatic vascular shunting: hepatic artery to portal vein, hepatic artery to hepatic vein, or portal vein to hepatic vein [8]. Complications include HOCF (primarily due to hepatic artery to hepatic vein shunting) and portal hypertension, encephalopathy, and biliary or mesenteric ischemia (from hepatic artery to portal vein or portal vein to hepatic vein shunting) [7–9]. HHT-related HOCF (HHT-HOCF) is often worsened by the anemia common in chronically bleeding HHT patients [9]. Treatment options for HHT-HOCF have been limited to supportive care (diuretics and correction of anemia) with liver transplantation being the only definitive treatment that reverses the high output state [10]. Hepatic artery embolization was attempted in the past, but the improvement was usually transient and it was associated with an extremely high rate of severe complications (hepatic necrosis, cholangitis) and death so it has been largely abandoned as a front-line treatment option [11].

Systemic anti-angiogenic therapies represent a promising new frontier in management of HHT-HOCF. These agents target and inhibit vascular endothelial growth factor (VEGF), a pro-angiogenic cytokine significantly elevated in HHT [12, 13]. The best-studied anti-angiogenic agent in HHT is systemic (intravenous) bevacizumab (Avastin®, Genentech, South San Francisco, CA), a humanized, recombinant monoclonal IgG antibody currently FDA-approved for the treatment of metastatic solid tumors. Bevacizumab binds to and neutralizes circulating VEGF [14]. Bevacizumab decreased cardiac output as well as reduced epistaxis and anemia in HHT-HOCF in a landmark phase II study of 25 patients [7], with normalization of cardiac index in 5 subjects and a partial response in 15 cases after 6 months of follow-up. Data suggests that disease manifestations return if bevacizumab is discontinued [14–16] so maintenance treatment is standard. There is no universally-accepted maintenance protocol. Despite the increasing worldwide use, there is very little published information on clinical use of bevacizumab use in HHT-HOCF, especially beyond the initial 6-month treatment period.

Given this paucity of data and lack of formal therapy guidelines, we conducted a multinational survey of HHT centers worldwide to best understand how bevacizumab was being used to manage HHT-HOCF and collect physician-reported outcomes of treatment. We hypothesized that treatment approaches would vary significantly between centers.

## Methods

### HHT centers and survey administration

This research was approved by the Institutional Review Board of the Mayo Clinic (approval number 14–006516). In January 2019 a 27-item electronic survey titled “Bevacizumab for High-Output Cardiac Failure in Hereditary Hemorrhagic Telangiectasia” was distributed to directors of all 26 North American HHT Centers of Excellence as well as 5 large international HHT centers in France, Norway, the Netherlands, Argentina, and Israel. This survey was conducted in partnership with the Cure HHT Foundation which is the primary advocacy group for HHT patients worldwide. HHT Centers of Excellence (established under the aegis of the Cure HHT Foundation) are large academic centers with demonstrated multidisciplinary expertise in the management of HHT patients [17]. The professional section of the online survey platform SurveyMonkey was used to host the survey and collect responses, utilizing page skip logic to guide responses through the survey based on prior answers and avoiding questions not applicable to the respondent. Utilizing this logic, each respondent was presented with 24 or 25 items in total.

Surveys were distributed by email 3 times: the first time to all 31 centers, and the second and third times to centers that had not yet responded. Paper versions of the survey were not sent. Centers not responding to the survey within 2 weeks of the final email were deemed non-responders.

### Survey content

The full survey is included in the Additional file 1. Respondents were instructed to include only patients treated with intravenous bevacizumab *primarily* for HOCF in HHT, not those treated primarily for HHT-related bleeding. The following domains were addressed in the survey: center location (1 item); total HHT-HOCF patients treated with bevacizumab (1 item); bevacizumab dosing strategy and protocol (9 items); treatment effectiveness and adverse events including discontinuation (5 items); use of other anti-angiogenic agents (2 items); prescriber characteristics (2 items); barriers to obtaining bevacizumab (1 item); and a 6 item case study designed to elucidate provider/center philosophy regarding the optimal threshold for initiation of systemic bevacizumab for a hypothetical patient with HHT-HOCF.

### Statistical analysis

All statistical analyses and figure preparation were performed using Microsoft Excel 2016 (Microsoft Corp., Redmond, WA).

## Results

### Respondent and survey completion data

A total of 23 physician center directors (19/26 North American centers and 4/5 International centers) responded to the survey for a 74% response rate. A total of 20 centers were included in the final dataset because 3 North American centers reported that they had not treated any HHT-HOCF patient with bevacizumab.

### Total patients treated

Thirty-five percent of centers reported treating less than 5 patients, 35% reported treating 5–10 patients, and 30% reported treating 11 or more HHT-HOCF patients with bevacizumab. In total, centers reported treating a minimum of 150 patients. This was calculated using the lower end of range-based answers if a definite number of patients was not provided (e.g. 11–15 patients counted as 11 patients).

### Bevacizumab induction and maintenance dosing protocols

During induction treatment (the series of doses given to all patients at the start of systemic bevacizumab treatment), all centers but one reported using a 5 mg/kg dose of bevacizumab every 2 weeks for either 4 doses (15% of centers) or 6 doses (80% of centers). One center reported administering 6 doses every 2 weeks followed by 4 doses every 4 weeks for a total of 10 induction doses.

Following induction, patients enter maintenance treatment (ongoing doses of bevacizumab given to prevent symptom recurrence). 55% of centers use a continuous maintenance strategy (regularly-scheduled bevacizumab maintenance doses given regardless of changes in cardiac output/cardiac index or HOCF symptomatology) and 45% use an intermittent (as needed) maintenance strategy (defined as bevacizumab follow-up doses given only as-needed for an increase in cardiac output/cardiac index or recurrence of HOCF symptoms).

For those using continuous maintenance, all centers utilized 5 mg/kg dosing and all but 3 utilized an every-4-week interval (intervals were every-6-week for 1 center and every-12-week for 2 centers). Three centers using continuous maintenance reported attempting to minimize overall bevacizumab exposure by lengthening the interval between treatment over time as tolerated by patients.

For those using intermittent maintenance, 5 mg/kg dosing was used by all but one center (which utilized 7.5 mg/kg dosing) and 56% utilized an every-2-week interval for 6 doses, although there was considerable variability in both interval (2 to 8 weeks) and number [1–6] of doses. Three centers additionally reported transitioning patients from intermittent maintenance to continuous maintenance if HOCF symptoms recurred or worsened recurrently while not receiving treatment.

### Bevacizumab effectiveness, adverse events, and discontinuation

**Fig. 1:**
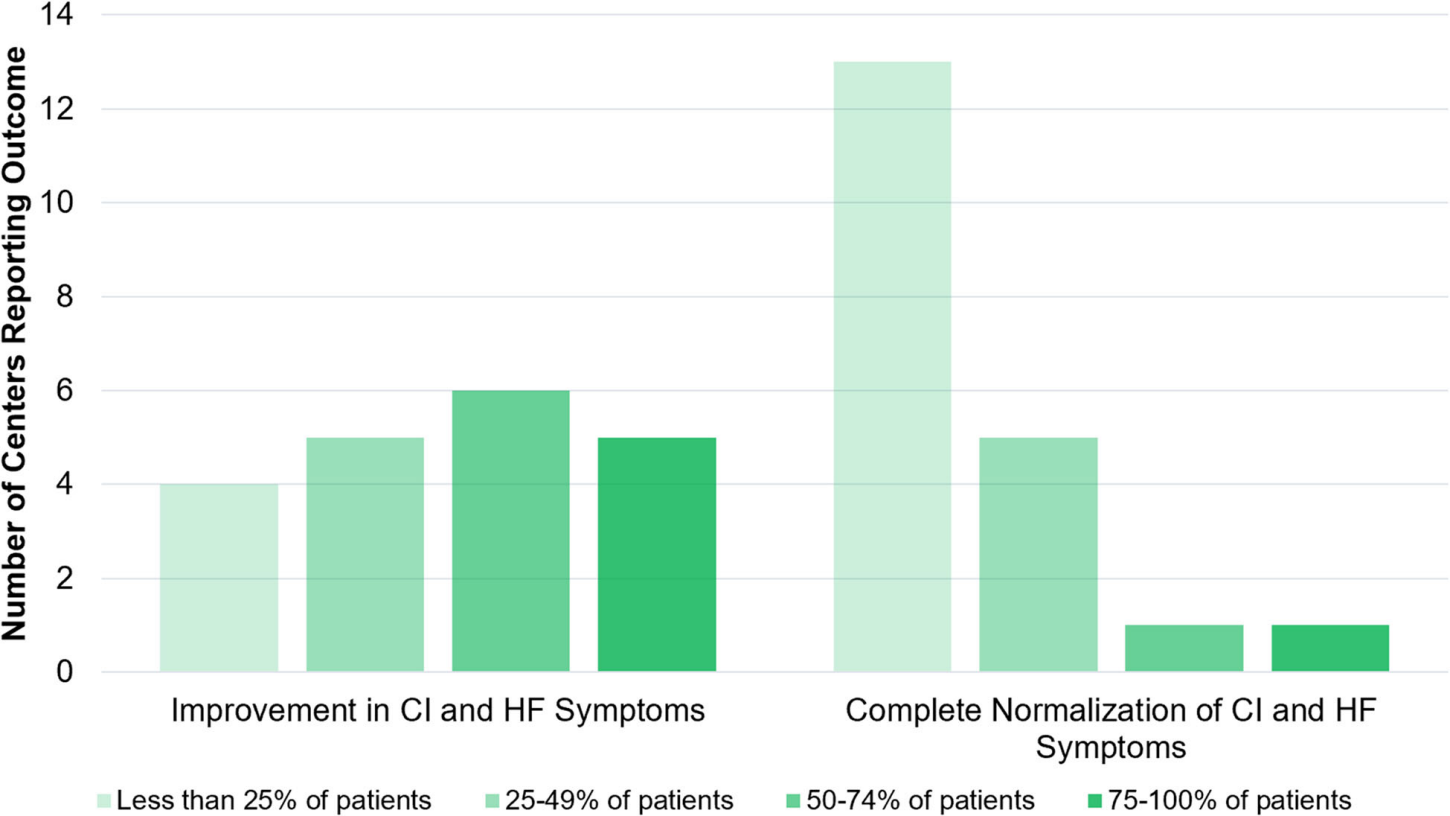
Reported efficacy of bevacizumab for HOCF by HHT centers. Bar color represents response selected by respondent (e.g. 75–100% of patients treated at their center had improvement in cardiac index and heart failure symptoms) and bar height reports the number of centers responding with that category for a given question. CI, cardiac index; HF, heart failure

Physician-reported outcomes regarding effectiveness of bevacizumab to manage HOCF symptoms were mixed. While a majority (55%) of centers reported that most patients treated achieved a significant improvement in cardiac index and HOCF symptoms, a significant minority (45%) reported that more than half of treated patients did not improve (Fig. 1). Indeed, only two centers reported that most treated patients achieved complete normalization of cardiac parameters, although one was a high-volume center (20 patients treated). Of note, this center reported a threshold for treatment corresponding to more mild disease (asymptomatic elevated cardiac output without chamber enlargement, see “Threshold for Initiation of Bevacizumab for HHT-HOCF” below and Table 1).

**Table 1:** Threshold for respondents to initiate bevacizumab for HOCF based on clinical scenario. CO, cardiac output; CI, cardiac index; NT-proBNP, N-terminal pro-brain natriuretic peptide

Clinical Scenario	Would Initiate Bevacizumab	Would Not Initiate Bevacizumab
Elevated CO/CI, normal NT-proBNP Moderate left atrial enlargement, no arrhythmias No signs or symptoms of HF or activity restriction	11%	89%
Elevated CO/CI, elevated NT-proBNP Moderate left atrial enlargement, no arrhythmias No signs or symptoms of HF or activity restriction	11%	89%
Elevated CO/CI, elevated NT-proBNP Severe left atrial enlargement, no arrhythmias No signs or symptoms of HF or activity restriction	26%	74%
Elevated CO/CI, elevated NT-proBNP Severe left atrial enlargement, paroxysmal atrial fibrillation No signs or symptoms of HF or activity restriction	63%	37%
Elevated CO/CI, elevated NT-proBNP Severe left atrial enlargement, paroxysmal atrial fibrillation Mild dyspnea on exertion, exercise limitation	79%	21%

**Fig. 2:**
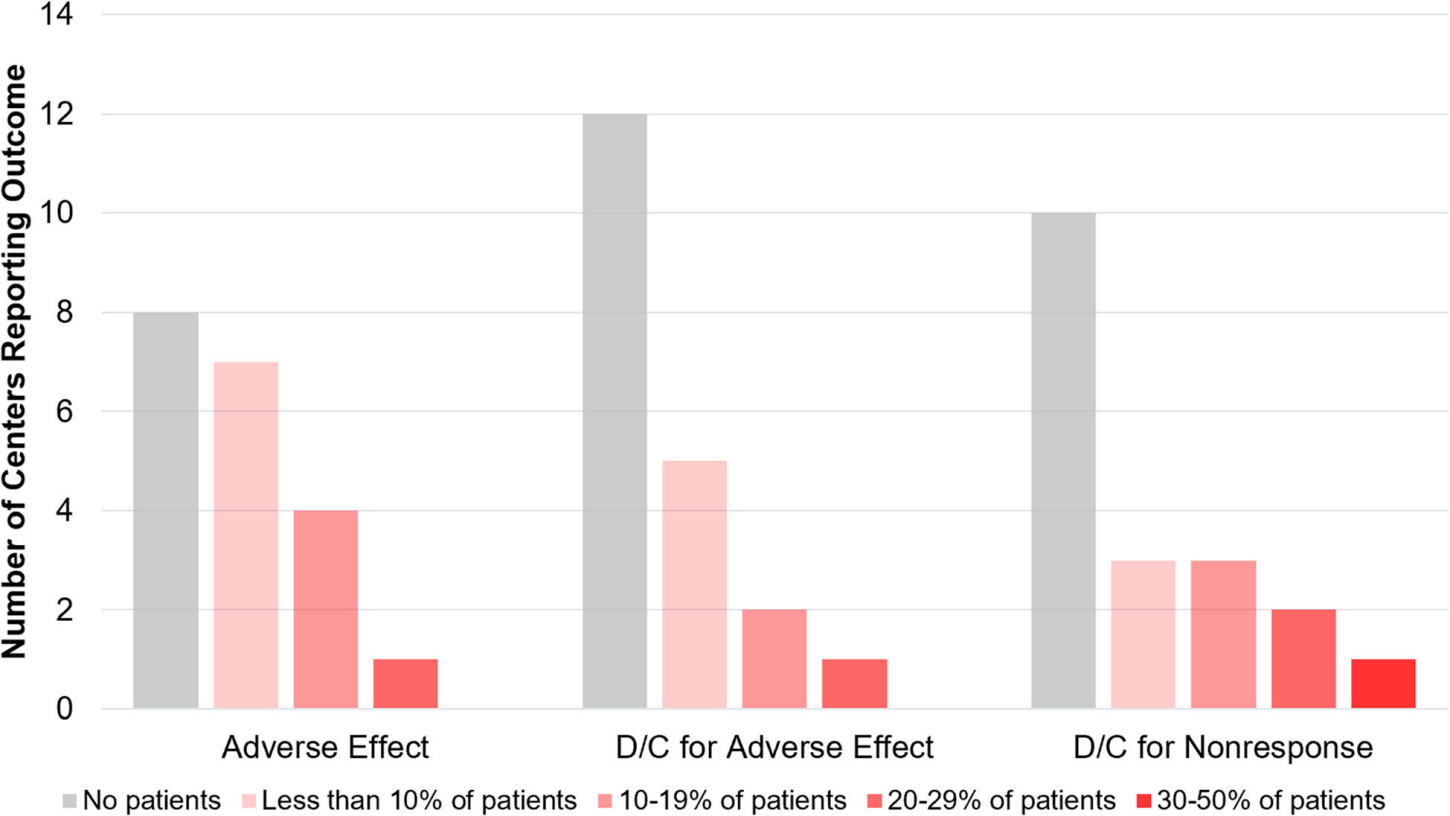
Rates of bevacizumab adverse effects (such as hypertension, proteinuria, or poor wound healing) and discontinuation reported by HHT centers. Bar color represents response selected by respondent (e.g. 10–19% of patients treated at their center had an adverse event) and bar height reports the number of centers responding with that category for a given question. One center that reported treating 2 patients reported that both had treatment discontinued for lack of response (not shown in figure)

Bevacizumab-associated adverse events, including new or worsening hypertension, renal dysfunction, proteinuria, and poor wound healing were uncommon (Fig. 2). All centers reported adverse event rates < 30% and three-quarters of centers reported adverse event rates < 10%. Most centers (60%) reported that no patient required treatment discontinuation due to adverse events, and 25% reported that such discontinuation occurred in < 10% of patients. Discontinuation for ineffectiveness was also uncommon: half of centers reported that no patient was discontinued for this reason, and 30% reported discontinuation for non-response in < 20% of patients.

### Threshold for initiation of Bevacizumab for HHT-HOCF

Provider threshold to prescribe bevacizumab for HOCF was assessed utilizing 6 items about management in the setting of a hypothetical HHT-related high cardiac output case (see full survey in Additional file 1). Nineteen of 20 centers provided answers to this case. Respondent answers are summarized in Table 1. Most centers reported not initiating bevacizumab for asymptomatic elevations in cardiac output/cardiac index or isolated left atrial enlargement; addition of an elevated N-terminal pro-brain natriuretic peptide (NT-proBNP) to the scenario did not change decision-making. Only after a hypothetical patient developed paroxysmal atrial fibrillation did most centers (63%) report they would initiate bevacizumab. Notably, 21% of centers reported they would not initiate bevacizumab for a patient with elevated cardiac output/cardiac index, severe left atrial enlargement, paroxysmal atrial fibrillation, and frank heart failure symptoms including dyspnea on exertion and exercise limitation. Additionally, 79% of centers reported that right heart catheterization is typically used to confirm findings of high cardiac output on echocardiogram prior to bevacizumab initiation.

### Prescriber characteristics

Systemic bevacizumab for HHT-HOCF is prescribed and managed primarily by hematologists (50% of centers) or pulmonologists (39% of centers), Table 2. Most centers (53%) report multidisciplinary input in the decision to initiate systemic bevacizumab. A single individual prescribed and managed bevacizumab in 42% of centers.

**Table 2:** Specialties of providers primarily prescribing and managing systemic bevacizumab for HHT-related HOCF in HHT centers

Specialty	Percent of Centers
Hematology	50%
Pulmonology	39%
Cardiology	11%
Gastroenterology	6%
Internal Medicine	6%

### Access to Bevacizumab

Seventy percent of centers report being able to obtain insurance coverage for bevacizumab to treat HOCF for most of their patients, but half reported it was a cumbersome process for most patients (Table 3). No center reported that high rates of insurance denial frequently led to administration of alternative treatments. Reported access was similar between U.S. and non-U.S. sites.

**Table 3:** Experience with insurance coverage issues and obtaining access to systemic bevacizumab for HOCF among HHT centers

Statement	Percent of Centers
Able to obtain insurance coverage for most or all patients	70%
Obtaining insurance coverage is a cumbersome process for most patients	50%
Have obtained bevacizumab using “Genentech Access to Care Foundation” compassionate use program	20%
Have had little or no trouble with Medicare approval of bevacizumab	35%
Able to obtain insurance coverage for oral anti-angiogenic therapies in cases of systemic bevacizumab insurance denial	5%
Frequently forced to search for alternative treatments because of insurance denial	0%

### Use of other systemic anti-Angiogenic agents

Use of other anti-angiogenic agents to treat HOCF in HHT aside from bevacizumab was uncommon. Three centers reported using pazopanib and one site each reported using tacrolimus and thalidomide; the remainder of centers (75%) reported using bevacizumab as the sole anti-angiogenic agent for the treatment of HHT-HOCF.

## Discussion

We present findings from the first large multinational survey study assessing the use of systemic bevacizumab in patients with HHT-HOCF. We report that bevacizumab is an effective, well-tolerated and widely-used treatment modality for patients with HHT-HOCF. We also report significant variation in dosing protocols, degree of treatment effectiveness and thresholds for treatment initiation among HHT Centers of Excellence. Given the lack of any FDA-approved medical therapies for HHT-HOCF, the findings of this survey, with its large cohort of patients, are likely to be of considerable interest to patients, providers and other stakeholders such as insurance providers and national health agencies.

Prior to this study, the only high-quality data describing IV bevacizumab use in HHT-HOCF was a landmark 2012 single-center phase II study of 25 patients aged 18–70 with who received systemic bevacizumab 5 mg/kg every 2 weeks for a total of 6 doses [7]. Of the 23 patients with 6-month follow-up data available, 5 patients had complete normalization of cardiac parameters (complete response) and 15 patients had significant improvement but not complete normalization (partial response). Given the rarity of HHT-HOCF and the fact that most centers likely initiated bevacizumab treatment after the publication of the phase II study, it is remarkable that ≥150 patients at 20 HHT centers have been treated with this agent. Systemic bevacizumab used for this indication was well-tolerated overall; side-effects were uncommon and discontinuation due to unacceptable side-effects was rare. This is consistent with other published safety data in HHT patients [18].

While overall precision is limited in a survey-based study, our study describes similar treatment effectiveness as the phase II study (Fig. 1). The prevailing consensus from HHT center directors was that most HHT-HOCF patients benefit from systemic bevacizumab and a minority achieve complete normalization of cardiac parameters and HOCF symptoms. The reasons for variations in therapeutic response need to be investigated and may relate to the size and overall burden of macrovascular arteriovenous connections between the hepatic artery and the hepatic vein. These large vascular channels are unlikely to respond to bevacizumab and may explain the lack of therapeutic response noted by many centers.

A majority of centers utilized an induction therapy regimen that mirrored the treatment regimen utilized in the previously described phase II study [7]. However, this study did not employ a maintenance dosing protocol. In contrast, our study shows that maintenance dosing is used in all responding HHT centers in some form. Given the overall paucity of data and total lack of well-controlled prospective studies assessing different maintenance dosing strategies, it is not surprising that maintenance dosing regimens varied widely. Patients at certain centers appear to be receiving substantially more bevacizumab overall as compared to patients at other centers. The consequences of this are unknown. Some described maintenance regimens appear to be extrapolated from the published literature describing use of bevacizumab for HHT-related bleeding [14–16, 19], while others likely reflect drug availability, cost considerations, or specific institutional practices. We certainly are not able to make specific recommendations on maintenance treatment based on the results of this study, which emphasize the need for well-controlled prospective studies to define the optimal maintenance strategy.

Prescriber characteristics and decision-making are additionally described in our study. Systemic bevacizumab for HOCF is most frequently prescribed by hematologists, perhaps reflective of the drug’s approval indications and widespread use as an antineoplastic agent and/or the considerable involvement of hematologists in the management of HHT-associated bleeding and iron deficiency anemia (which is also treated with bevacizumab). Pulmonologists were the second-most frequent prescribers of bevacizumab likely owing to their involvement in overall management of cardiopulmonary disease in HHT. Most centers involve multiple specialties in the overall discussion about proceeding with bevacizumab initiation in a patient, and per the prevailing opinion of center directors who responded to our survey, currently most patients are initiated on the drug only after they develop cardiac complications such as atrial fibrillation or symptoms of heart failure decompensation. The fact that the threshold for bevacizumab initiation is later in the course of these patients for most centers may reflect a possible benefit for more input from cardiology specialists in making this determination, although cardiologists with expertise in HHT-HOCF are uncommon. Initiation of treatment in more advanced disease may also impact outcomes of treatment; one very high volume center that reported most patients normalizing cardiac output with bevacizumab also reported treating earlier in the disease course. More sensitive echocardiographic indices, such as diastolic function or assessment for global longitudinal strain with speckle-tracking echocardiography could potentially identify patients earlier in the course of disease that could benefit from bevacizumab. Additionally, 21% of centers reported they would not initiate bevacizumab to treat advanced HOCF, presumably relying entirely on symptomatic heart failure management as a bridge to possible liver transplantation. Variation in practice may reflect respondent specialty differences, the overall paucity of high-quality data supporting the use of bevacizumab for HHT-HOCF (including the lack of a randomized study), and the frequent and inappropriate classification of bevacizumab as a cytotoxic chemotherapy agent despite its targeted mechanism of action and mild side-effect profile. Certainly, access to bevacizumab may also play a role: half of centers reported that obtaining insurance approval for patients was difficult, although no center reported that frequent insurance denial commonly resulted in prescription of an alternative treatment.

Our study had several notable limitations owing primarily to its survey-based nature. These include reporter bias, reporter inaccuracy, and recall bias, among other potential issues. Detailed patient data were not obtained. Ranges were used for several questions with numeric answers to facilitate ease of response, but this prevented aggregation of precise numbers and limited the statistical analysis. We attempted to follow best practices in survey design [20], but our survey was unique and not a previously validated instrument.

In conclusion, most North American HHT Centers of Excellence are utilizing bevacizumab to manage HHT-HOCF, with treatment effectiveness and safety approximating that of the best published data describing its use for this indication. Bevacizumab is administered primarily by hematologists and pulmonologists, with some insurance-related access limitations. Induction dosing strategies appear to be fairly uniform but maintenance strategies have considerable variability between centers. The findings of this study highlight the need for well-controlled studies to address knowledge gaps in the use of systemic bevacizumab to treat HHT-HOCF.

## Supplementary information

**Additional file 1.** Paper version of electronic survey sent to HHT centers.

## Data Availability

Please email Hanny Al-Samkari at hal-samkari@mgh.harvard.edu for original data.

## Abbreviations

AVMs: Arteriovenous malformations CO Cardiac output
CI: Cardiac index
GI: Gastrointestinal
HF: Heart failure
HHT: Hereditary hemorrhagic telangiectasia HOCF High-output cardiac failure HHT-HOCF Hereditary hemorrhagic telangiectasia-associated high-output cardiac failure
NT-proBNP: N-terminal pro-brain natriuretic peptide

## References

[1] Fuchizaki Uichiro, Miyamori Hirotoshi, Kitagawa Shunsuke, Kaneko Shuichi, Kobayashi Kenichi (2003). Hereditary haemorrhagic telangiectasia (Rendu-Osler-Weber disease). The Lancet.

[2] Letteboer TG, Mager HJ, Snijder RJ (2008). Genotype-phenotype relationship for localization and age distribution of telangiectases in hereditary hemorrhagic telangiectasia. Am J Med Genet A.

[3] Abdalla SA, Geisthoff UW, Bonneau D (2003). Visceral manifestations in hereditary haemorrhagic telangiectasia type 2. J Med Genet.

[4] Kritharis A, Al-Samkari H, Kuter DJ (2018). Hereditary hemorrhagic telangiectasia: diagnosis and management from the hematologist's perspective. Haematologica.

[5] Al-Samkari H, Kritharis A, Kuter DJ (2018). Infections and vaccination in hereditary hemorrhagic telangiectasia: microbiological evidence-based considerations. Haematologica.

[6] Buonamico P, Suppressa P, Lenato GM (2008). Liver involvement in a large cohort of patients with hereditary hemorrhagic telangiectasia: Echo-color-Doppler vs multislice computed tomography study. J Hepatol.

[7] Dupuis-Girod S, Ginon I, Saurin JC (2012). Bevacizumab in patients with hereditary hemorrhagic telangiectasia and severe hepatic vascular malformations and high cardiac output. JAMA.

[8] Clinical Practice Guidelines EASL (2016). Vascular diseases of the liver. J Hepatol.

[9] Garcia-Tsao G, Korzenik JR, Young L (2000). Liver disease in patients with hereditary hemorrhagic telangiectasia. N Engl J Med.

[10] Faughnan ME, Palda VA, Garcia-Tsao G (2011). International guidelines for the diagnosis and management of hereditary haemorrhagic telangiectasia. J Med Genet.

[11] Garg N, Khunger M, Gupta A, et al. Optimal management of hereditary hemorrhagic telangiectasia. J Blood Med. 2014;5:191–206. 10.2147/JBM.S45295 PMC4206399 25342923

[12] Cirulli A, Liso A, D’Ovidio F (2003). Vascular endothelial growth factor serum levels are elevated in patients with hereditary hemorrhagic telangiectasia. Acta Haematol.

[13] Sadick H, Riedel F, Naim R (2005). Patients with hereditary hemorrhagic telangiectasia have increased plasma levels of vascular endothelial growth factor and transforming growth factor-beta1 as well as high ALK1 tissue expression. Haematologica.

[14] Iyer VN, Apala DR, Pannu BS (2018). Intravenous Bevacizumab for refractory hereditary hemorrhagic telangiectasia-related epistaxis and gastrointestinal bleeding. Mayo Clin Proc.

[15] Al-Samkari H, Kritharis A, Rodriguez-Lopez JM (2019). Systemic bevacizumab for the treatment of chronic bleeding in hereditary haemorrhagic telangiectasia. J Intern Med.

[16] Guilhem A, Fargeton AE, Simon AC (2017). Intra-venous bevacizumab in hereditary hemorrhagic telangiectasia (HHT): a retrospective study of 46 patients. PLoS One.

[17] Cure HHT Foundation. Criteria for a North American Cure HHT Center of Excellence, 2017.

[18] Buscarini E, Botella LM, Geisthoff U (2019). Safety of thalidomide and bevacizumab in patients with hereditary hemorrhagic telangiectasia. Orphanet J Rare Dis.

[19] Al-Samkari H (2019). Systemic Bevacizumab for hereditary hemorrhagic telangiectasia: considerations from observational studies. Otolaryngol Head Neck Surg.

[20] Draugalis JR, Coons SJ, Plaza CM (2008). Best practices for survey research reports: a synopsis for authors and reviewers. Am J Pharm Educ.

