# Supplementary material for: Systemic bevacizumab for high-output cardiac failure in hereditary hemorrhagic telangiectasia: an international survey of HHT centers
Source: Orphanet J Rare Dis. 2019 Nov 14;14:256. doi: 10.1186/s13023-019-1239-6 (PMC6857247; doi:10.1186/s13023-019-1239-6)
Supplement: Supplementary file 1 — Additional file 1. Paper version of electronic survey sent to HHT centers. [file 13023_2019_1239_MOESM1_ESM.pdf]

***Supplemental Index***

*Note: The following survey was administered electronically in this study. This document attempts to duplicate the survey with the page skip logic present in the actual survey noted in capital text in parentheses.*

# Bevacizumab for High-Output Cardiac Failure in Hereditary Hemorrhagic Telangiectasia

**This questionnaire pertains to intravenous bevacizumab treatment PRIMARILY for the treatment of hereditary hemorrhagic telangiectasia-related high output cardiac failure (HOCF). Do not include patients that are being treated with IV bevacizumab primarily for HHT-related epistaxis or gastrointestinal bleeding.**

*Aims of this survey:*

*1. Understand local practice variations regarding dosing of bevacizumab (both for initial 'induction' as well as for 'maintenance' dosing).*

*2. Assess the safety and efficacy of IV bevacizumab treatment for HHT-related HOCF.*

*3. Assess use of other anti-angiogenic agents for HHT-related HOCF.*

* 1. Please enter the name of your HHT Center here:

* 2. How many patients with HHT-related HOCF have you treated with IV bevacizumab?

- ❍ 5 or fewer
- ❍ 6-10
- ❍ 11-15
- ❍ >15
- ❍ Please provide the exact number if this is known:

## Dosing and Administration of Bevacizumab

*The next set of questions focus on your center's protocol with regards to 'induction' as well as 'maintenance' dosing of bevacizumab for HHT-related HOCF.*

*Definition of induction dosing: This refers to the usual series of doses given to every patient at the start of treatment (for example, 5 mg/kg IV every 2 weeks for a total of 6 doses).*

* 3. What is the dose of bevacizumab used for induction treatment?

- ❍ 2.5 mg/kg
- ❍ 5 mg/kg
- ❍ 7.5 mg/kg
- ❍ 10 mg/kg
- ❍ Other (please specify):

* 4. What is the interval between doses during induction treatment?

- ❍ 1 week
- ❍ 2 weeks
- ❍ 3 weeks
- ❍ 4 weeks
- ❍ Other (please specify):

* 5. How many doses are given during the induction treatment period?

- ❍ 4
- ❍ 6
- ❍ 8
- ❍ Other (please specify):

6. If your induction treatment dosing schedule does not conform to the options given above (e.g. dose or interval changes over the course of induction), please describe it here, providing all details.

*Definition of maintenance dosing: This refers to additional doses of bevacizumab given after the completion of induction dosing in order to prevent HOCF recurrence.*

* 7. The common maintenance strategies used at various centers are described below. Please select the strategy that best fits your practice. After you select an option, you will be taken to a page that will allow you to clarify the specifics of how you treat patients.

- ❍ Continuous maintenance dosing strategy: This refers to regularly scheduled bevacizumab maintenance doses given after completion of induction dosing irrespective of cardiac index or clinical symptoms. Example: a patient who has been set-up to receive an infusion of 5 mg/kg bevacizumab every 4 weeks on a regular schedule after completion of induction dosing. (UPON SELECTION, RESPONDENT IS AUTOMATICALLY SKIPPED TO QUESTION 8)

- ❍ Intermittent (as-needed) dosing strategy: This refers to bevacizumab doses only given as-needed for an increase in cardiac index or a recurrence of heart failure symptoms. Example: a patient has completed 6 induction doses and is now followed with quarterly echocardiograms. After 6 months of follow up, she is found to have a significant increase in cardiac index and receives 2 additional doses of bevacizumab, 5 mg/kg, 2 weeks apart. She then resumes quarterly echocardiograms and will receive further bevacizumab doses if she again has an increase in cardiac index or recurrence of heart failure symptoms. (UPON SELECTION, RESPONDENT IS AUTOMATICALLY SKIPPED TO QUESTION 10)

* 8. What is the typical continuous maintenance regimen you use? Enter more than one regimen if you routinely use multiple different continuous maintenance regimens. (RESPONDENTS ARE GIVEN SPACE TO DESCRIBE UP TO 3 CONTINUOUS MAINTENANCE REGIMENS, SPECIFYING DOSE AND INTERVAL, WITH THE OPTION TO FREE-TEXT IF DESIRED)

9. If you modify your continuous maintenance dosing or dose interval over time, please describe how you do this in the text box below. Example: Patient receives 5 mg/kg every 4 weeks for the first year of continuous maintenance, and if heart failure symptoms do not recur, the dose/interval is modified, e.g. to 2.5 mg/kg every 4 weeks or 5 mg/kg every 8 weeks. (UPON SELECTION, RESPONDENT IS AUTOMATICALLY SKIPPED TO QUESTION 12)

* 10. After a patient "triggers" the need for maintenance bevacizumab, what is the typical dosing used for intermittent maintenance? Enter more than one regimen if you routinely use multiple different intermittent maintenance regimens. (RESPONDENTS ARE GIVEN SPACE TO DESCRIBE UP TO 3 INTERMITTENT MAINTENANCE REGIMENS, SPECIFYING DOSE, INTERVAL, AND NUMBER OF DOSES ADMINISTERED, WITH THE OPTION TO FREE-TEXT IF DESIRED)

11. If you modify your intermittent maintenance regimen over time, please describe how you do this in the text box below. Example: Some providers may utilize a "hybrid strategy" where the patient starts with intermittent maintenance initially and then moves on to a personalized regularly-scheduled infusion strategy once the individual's bevacizumab needs become clearer with longer follow-up.

**Bevacizumab Effectiveness, Adverse Events and Discontinuation**

* 12. Approximately what percentage of patients experienced a significant improvement (but not complete normalization) in cardiac index and heart failure after initiation of bevacizumab treatment?

- Less than 25%
- 25-49%
- 50-74%
- 75%-100%

* 13. Approximately what percentage of patients experienced complete normalization of cardiac index and resolution of heart failure symptoms after initiation of bevacizumab treatment?

- Less than 25%
- 25-49%
- 50-74%
- 75%-100%

* 14. Approximately what percentage of patients experienced a significant adverse effect (new or worsening hypertension, renal dysfunction, proteinuria, poor wound healing, etc.) after bevacizumab treatment?

- No patient met this criterion
- Less than 10%
- 10-19%
- 20-29%
- 30-50%

Other (please specify)

* 15. Approximately what percentage of patients required discontinuation of bevacizumab due to a significant side effect (new or worsening hypertension, renal dysfunction, proteinuria, poor wound healing, etc.)?

- ❍ No patient met this criterion
- ❍ Less than 10%
- ❍ 10-19%
- ❍ 20-29%
- ❍ 30-50%
- ❍ Other (please specify)

* 16. Approximately what percentage of patients required discontinuation of bevacizumab treatment due to complete non-response (no improvement in cardiac index or heart failure symptoms)?

- ❍ No patient met this criterion
- ❍ Less than 10%
- ❍ 10-19%
- ❍ 20-29%
- ❍ 30-50%
- ❍ Other (please specify)

**Use of Other Anti-Angiogenic Agents**

* 17. Are you currently using any oral anti-angiogenic agent to treat HHT-related HOCF? Select all that you are currently using or have previously used.

- ❑ Thalidomide
- ❑ Pomalidomide
- ❑ Pazopanib
- ❑ Tacrolimus

❑ I am not currently and have not previously used any oral anti- angiogenic agents to treat HHT-related HOCF

❑ Other (please specify)

* 19. Approximately how many patients have you transitioned from IV bevacizumab to a different anti- angiogenic agent due to non-response or partial (inadequate) response?

❍ None

❍ 1-2 patients

❍ 3-4 patients

❍ 5-6 patients

❍ Other (please specify)

**Insurance Considerations for Use of Systemic Bevacizumab**

20. Please select one or more statements from the list below that accurately represent your center's experience with obtaining insurance coverage for IV bevacizumab for HHT-related HOCF.

❑ I have been able to obtain insurance coverage for most or all patients for whom I have prescribed IV bevacizumab

❑ I have had little to no trouble getting Medicare (age >65) to cover IV bevacizumab

❑ I am frequently forced to search for alternative treatments because of high rates of insurance denial for IV bevacizumab

❑ Obtaining insurance coverage for bevacizumab is a cumbersome process for most of my patients (requiring insurance prior authorization, peer-to-peer review, letters of appeal, etc.)

❑ I have used the "Genentech Access to Care Foundation" program to obtain IV bevacizumab for patients who have been denied insurance coverage

❑ I have been able to obtain insurance coverage for oral anti-angiogenic therapies in cases where IV bevacizumab was denied

**Clinical Indication for Bevacizumab Initiation**

*Please consider the following clinical case and questions.*

*A 54-year-old female with a family history of the ACVRL1 mutation (HHT-2) presents for initial HHT screening and workup. She is asymptomatic except for some minor nosebleeds. She engages in moderate to heavy aerobic exercise (cycling classes) at the gym several days a week and reports no shortness of breath or any kind of activity limitation.*

*Liver CT demonstrates diffuse HHT-related vascular changes including AVMs and telangiectasias along with enlargement of the main hepatic artery (8 mm diameter).*

*Echocardiography is notable for the following:*

*1. Elevated cardiac output (10.2 Liters/min) with a cardiac index of 5.1 liters/minute/meters squared BSA (normal 2.5- 4.2)*

*2. Mild enlargement right atrium and moderate to severe enlargement of the left atrium.*

*3. Normal RV and LV size and systolic function.*

*Hemoglobin is 13 gm/dL. The NT-proBNP is normal (250 pg/ml; normal < 300 pg/ml). The rest of her screening studies including ferritin, liver and kidney function as well as brain MRI and chest CT are all normal. Physical examination shows some mild jugular venous dilatation along with a hepatic bruit (best heard over the midline in the subxiphoid area).*

*There is no evidence for lower extremity edema, ascites, hepatomegaly or crackles on lung auscultation.*

* 20. If this patient was being evaluated at your HHT center, would you advise initiation of IV bevacizumab this point in time to treat the elevated cardiac output and atrial chamber enlargement in this otherwise asymptomatic patient?

❍ Yes, we would typically treat patients like her proactively to prevent the development of overt heart failure and other complications. (UPON SELECTION, RESPONDENT IS AUTOMATICALLY SKIPPED PAST QUESTION 21 TO THE NEXT VIGNETTE)

❍ No, we would not start IV bevacizumab at this time, but would follow her closely and initiate treatment only when symptomatic heart failure develops (dyspnea, lower extremity edema, orthopnea, paroxysmal nocturnal dyspnea, ascites, etc.) (UPON SELECTION, RESPONDENT IS AUTOMATICALLY SKIPPED TO QUESTION 21)

❍ Other (please specify) (UPON SELECTION, RESPONDENT IS AUTOMATICALLY SKIPPED TO QUESTION 21)

21. Would your decision change if this patient was found to have a mildly elevated N-terminal pro-brain natriuretic peptide (NT-proBNP) of 450 pg/ml (normal <300 pg/ml)?

- ❍ Yes, IV bevacizumab would be appropriate at this time given the elevated NT-proBNP.
- ❍ No, this does not change my decision; I still would not start IV bevacizumab at this time.
- ❍ Other (please specify)

*The patient was not started on IV bevacizumab. She presents for a follow-up echocardiogram after 9 months. This study shows a slight increase in the size of both atria and the left atrial enlargement is now classified as severe (previously moderate to severe). She is still asymptomatic and is participating in regular aerobic exercise as previously described.*

* 22. Would you advise initiation of IV bevacizumab at this time in this otherwise asymptomatic patient with elevated cardiac output and progressive atrial chamber enlargement?

- ❍ Yes
- ❍ No
- ❍ Other (please specify)

*The patient was not started on IV bevacizumab and was advised a follow-up echocardiogram after 6 months. She now presents 3 months later (12 months after initial assessment) with intermittent episodes of palpitations that started about a month ago. She was evaluated at a local emergency room and was found to be in paroxysmal atrial fibrillation during one of these episodes. She has had 4-5 such episodes in the last month. A repeat echocardiogram appears to be unchanged from the previous findings noted 6 months ago and the patient is in sinus rhythm.*

* 23. Would you advise initiation of IV bevacizumab in this HHT patient with high cardiac output state, atrial chamber enlargement and paroxysmal atrial fibrillation?

- ❍ Yes
- ❍ No
- ❍ Other (please specify)

*The patient was not started on IV bevacizumab and presents 6 months later (18 months after initial assessment) for follow up. She now reports mild dyspnea on exertion and has slightly slowed the pace of her cycling. She continues to have intermittent brief episodes of paroxysmal atrial fibrillation (once every 2 months).*

*Physical examination and laboratory values are unchanged, but the echocardiogram now shows the following changes:*

*1. Elevated cardiac output is now 11 L/min (previously 10.2 liters/min) with a cardiac index of 5.5 L/min/m2 BSA (previously 5.1 liters/min/m2 BSA; normal 2.5-4.2).*

*2. Mild enlargement right atrium and severe enlargement of the left atrium (slight further enlargement when compared to previous echo 6 months ago).*

*3. Normal RV and LV size and systolic function.*

* 24. Would you advise initiation of IV bevacizumab in this patient with mild dyspnea, progression of atrial chamber enlargement and paroxysmal atrial fibrillation?

- Yes
- No
- Other (please specify)

* 25. In your center, do you typically use a right heart catheterization (RHC) to confirm findings of high cardiac output on echocardiogram? This question assumes that the echo does not show other abnormalities that might warrant a RHC (e.g. significant pulmonary hypertension).

- Yes, we typically perform a RHC in all patients being considered for IV bevacizumab therapy for HOCF.
- No, we typically do not perform a right heart catheterization and are comfortable starting IV bevacizumab based on the echo findings alone.
- Other (please specify)

**Prescriber Characteristics**

* 26. At your center, who typically prescribes IV bevacizumab for HHT-related HOCF (select one or more responses from the list below)?

- ❑ There is a single individual (please indicate specialty in the next question) who typically takes the lead regarding the initiation and subsequent dosing of IV bevacizumab for HHT-related HOCF.
- ❑ There are multiple individuals all from the same specialty (please indicate specialty in the next question) who typically take the lead regarding the initiation and subsequent dosing of IV bevacizumab for HHT-related HOCF.
- ❑ The decision to initiate IV bevacizumab is based on the consensus of a team of HHT experts that may include a hematologist, cardiologist, hepatologist, and pulmonologist, among others.
- ❑ Other (please specify)

* 27. Please indicate the primary specialty(s) of the individual(s) involved in prescribing IV bevacizumab treatment for HHT-related HOCF. Select more than one option if appropriate.

- ❑ Hematology
- ❑ Cardiology
- ❑ Pulmonology
- ❑ Gastroenterology
- ❑ Interventional radiology
- ❑ Other (please specify)
